# DUCK‐Net: automated deep learning segmentation of ductular reactions in murine liver injury captures multicellular niche dynamics from H&E morphology

**DOI:** 10.1002/path.70113

**Published:** 2026-09-08

**Authors:** Nathalie Feeley, Kai Williams, Caitlin McCaffrey, Daniel Field, Kyle Davies, Hugh Warden, Luke Boulter, Steve Thorn, Tak Yung Man, Wei‐Yu Lu, Stephen J Wigmore, Ewen M Harrison, Stuart J Forbes, Ian P Tomlinson, Timothy J Kendall, Rachel V Guest

**Affiliations:** ^1^ Institute of Genetics and Cancer University of Edinburgh Edinburgh UK; ^2^ Department of Oncology University of Oxford Oxford UK; ^3^ Institute of Regeneration and Repair University of Edinburgh Edinburgh UK; ^4^ Department of Clinical Surgery University of Edinburgh Edinburgh UK; ^5^ Centre for Medical Informatics, Usher Institute University of Edinburgh Edinburgh UK; ^6^ Edinburgh Pathology University of Edinburgh Edinburgh UK

**Keywords:** artificial intelligence, image segmentation, whole‐slide imaging, ductular reaction, cholestasis, liver regeneration, deep learning

## Abstract

Ductular reactions (DRs) are dynamic and complex multicellular responses that occur as a result of various hepatic injuries. Precise identification and quantification of the extent of DRs is a cornerstone of pre‐clinical modelling of liver disease, with links to inflammation, fibrosis, regeneration, and disease severity. Here, we apply a deep learning model, Deep Understanding Convolutional Kernel (DUCK‐Net), to the automated detection and segmentation of DRs in whole‐slide histopathological images of murine models of liver damage. Following annotation of a training dataset by a specialist liver histopathologist, we demonstrate accelerated performance and accurate detection, achieving a mean Dice coefficient (model‐expert segmentation overlap) of 85.4% and a specificity of 98%, indicating minimal false positives. Evaluation of model validity and utility was achieved with a histological time course of cholestatic injury and recovery using 3,5‐diethoxycarbonyl‐1,4‐dihydrocollidine diet (DDC) in mice. When assessed against a multiple linear regression model incorporating core epithelial and stromal components of the DR as quantified using immunohistochemistry (IHC), DUCK‐Net predicted the spatiotemporal response to injury and repair/resolution with a coefficient of determination (*R* ^2^) of 0.88. Moreover, DUCK‐Net kinetics strongly correlated with published spatial transcriptomic (Stereo‐seq) analysis of the DDC model, demonstrating that H&E‐based segmentation captured molecular DR dynamics comparable to or exceeding that of individual IHC markers without the need for immunostaining. DUCK‐Net provides a novel and accessible platform for rapid, accurate histological quantification of liver injury reflective of the matrix‐rich, multicellular regenerative niche observed in DRs. © 2026 The Author(s). *The Journal of Pathology* published by John Wiley & Sons Ltd on behalf of The Pathological Society of Great Britain and Ireland.

## Introduction

Cholestatic liver diseases such as primary sclerosing cholangitis (PSC), primary biliary cholangitis (PBC), and other cholangiopathies are associated with the expansion of periportal ductular cells, known histologically as the ductular reaction (DR) [1, 2]. In addition to the proliferation and/or hyperplasia of biliary epithelial cells (BECs) and hepatic progenitor cells (HPCs), DRs are characterised by fibrogenesis and infiltration of stromal and inflammatory cells including macrophages into the periportal regions [3]. DR expansion has been shown to be accompanied by angiogenesis [4] and, in certain contexts, transdifferentiation of hepatocytes [5, 6]. Deposition of extracellular matrix (ECM), in particular laminin, within the DR maintains a local ‘niche’ to determine the cell fate of HPCs and modify the cellular response according to injury aetiology [7]. Further to the well‐established association with biliary injury, DRs can also be observed in the context of severe hepatocyte damage, including alcoholic hepatitis and metabolic dysfunction‐associated liver disease (MASLD), and the extent of DR can act as a histological correlate of disease severity and patient mortality [3, 8, 9].

Dietary 3,5‐diethoxycarbonyl‐1,4‐dihydro‐collidine (DDC) in mice recapitulates the effects of human cholestatic disease by inducing periportal injury through the induction of interlobular biliary obstruction with protoporphyrin plugs. Toxic accumulation of bile acids activates BECs and initiates periportal inflammation and fibrosis, as well as secondary hepatocyte damage, as evidenced by raised serum transaminases and parenchymal necrosis [10, 11]. BECs upregulate genes that trigger recruitment and differentiation of macrophages, further stimulating BEC proliferation, as well as inducing HPC differentiation into BECs and dampening the proliferation of periportal hepatocytes [12]. The consistent induction of periportal DRs by DDC within weeks of feeding and the resultant recovery following dietary withdrawal have made it a frequently used model of liver injury and regeneration, and its spatiotemporal dynamics have been widely studied [12].

The quantification of DRs within histopathological slides of murine liver tissue is a complex and time‐consuming process subject to inter‐observer variability. A manual approach to annotation requires histopathology experience, creates a bottleneck in research workflow, and introduces inconsistencies in murine studies. Recent advances in deep learning models have facilitated the automation of image analysis in medical research. Computer vision (the interpretation of visual information by machines) and in particular image segmentation have showcased the ability to identify complex structures in medical images. Models such as U‐Net, Mask R‐CNN, and DeepLab have been developed specifically for the detection of nuclei, cells, and complex tissue boundaries or other multi‐scale features within histological slides [13, 14, 15]. Deep Understanding Convolutional Kernel (DUCK‐Net), a specialised neural network architecture, was previously developed and applied to the segmentation of human colonic polyps, enabling the automated detection and location of polyp borders [16]. This convolutional neural network (CNN) combines the encoder‐decoder structure of U‐Net, an existing deep learning architecture, and a custom‐designed convolutional block with residual down sampling. DUCK‐Net uses six variations of convolutional blocks in parallel, allowing the network to adaptively select the most effective features during training. Low‐level detail is preserved by the addition of a secondary U‐Net downscaling layer that bypasses image processing. It has been proposed that the DUCK‐Net architecture improves on previous models through the accurate prediction of border location, with the ability to utilise initial information at each resolution level in the encoder segment, allowing the model to learn effectively from small datasets [16].

We hypothesised that this multi‐resolution approach might be well suited for histological images where cellular structures vary in size and appearance, allowing complex segmentation of heterogenous multicellular regions, such as those seen in liver injury, and the detection of subtle differences between cell populations. The use of data augmentation allows DUCK‐Net to handle variability in tissue staining and preparation, common challenges in histological analysis of slides of liver injury. In this study, we implemented DUCK‐Net for the production of annotations of DRs present in murine livers treated with DDC diet and evaluate its predictive accuracy to the ground truth annotation. To provide orthogonal validation of H&E segmentation using DUCK‐Net to accurately capture the complex multi‐component DR, we present correlation analyses with core classical immunohistochemistry (IHC) markers as well as molecular data using a previously published, publicly available spatial transcriptomics dataset (Stereo‐seq) from an independent DDC study [12].

## Materials and methods

### Ethical approval

All animal work included in this study was approved by the University of Edinburgh Animal Welfare and Ethics Board (PPL Nos. PP3266462, PPD31D3D4) in accordance with the UK Animals (Scientific Procedures) Act 1986. See Materials & Methods for full details.

### Animal experiments

C57BL/6 mice were bred in‐house at the University of Edinburgh, UK, and housed in pathogen‐free conditions. At 6–8 weeks old, female mice were fed 0.1% 3,5‐diethoxycarbonyl‐1,4‐dihydro‐collidine for up to 21 days and placed back on standard chow for the recovery model. Four mice were culled at six timepoints. Mice fed normal chow were used as a baseline control group. Sample size was calculated *a priori* based on published Krt19+ area quantification data from Fickert *et al* in the DDC model [17]. Assuming group means of 5 (control), 8 (peak injury), and 6 (recovery) arbitrary units with a pooled standard deviation (SD) of 1, the effect size (Cohen's *f*) was 1.25. Using one‐way ANOVA with *α* = 0.05% and 80% power, *n* = 4 mice per group were deemed to be required, providing 91% power to detect significant differences across the injury‐recovery time course. In total, 24 mice were bred specifically for the DDC time course reported in this study. A further 61 archival H&E‐stained sections each from independent mice that had undergone 21 days of DDC dietary injury as control groups in other in‐house studies were included for DUCK‐Net training (one whole‐slide image), validation (50 whole‐slide images), and testing (10 whole‐slide images). For cross‐model generalisation, archival H&E‐stained sections were obtained from mice subjected to two further models of liver injury: a methionine‐choline‐deficient (MCD) diet for 2 weeks (*n* = 9) and a choline‐deficient diet supplemented with 0.15% ethionine in the drinking water [choline‐deficient, ethionine‐supplemented (CDE) diet] for 6 weeks followed by 2 weeks of recovery on standard chow (*n* = 6) [18, 19].

### Immunohistochemistry and slide analysis

Mice were culled using a Schedule 1 procedure, and livers were perfused with PBS via the inferior vena cava. Livers were fixed overnight in 10% buffered formalin before paraffin embedding, cutting 4‐μm sections and staining with H&E. Sections were deparaffinised in xylene and rehydrated through serial alcohols. The following antibodies were used: cytokeratin 19 (KRT19) (TROMA‐III) (Developmental Studies Hybridoma Bank, Iowa City, IA, USA; AB 21335570), SRY‐Box Transcription Factor 9 (SOX9) (Sigma‐Aldrich, St. Louis, MO, USA; HPA001758), epithelial cell adhesion molecule (EpCAM) (Cell Signaling Technology, Danvers, MA, USA; 93790T), and α‐smooth muscle actin (α‐SMA) (Sigma‐Aldrich; A2547). Heat‐induced epitope retrieval was performed through immersion in Tris‐EDTA buffer (10 mm Tris, 1 mm EDTA, pH9.0) for 10 min before staining. Immunoreactivity was visualised using 3,3‐diaminobenzidine (DAB) substrate solution with hydrogen peroxide in phosphate‐buffered saline (PBS) following the manufacturer's protocol (Dako, Glostrup, Denmark). To stain pan‐collagen fibres, sections were incubated with Picrosirius Red (PSR) solution (0.1% Sirius Red in saturated picric acid) for 60 min at room temperature followed by rinsing with 0.5% acetic acid solution.

Whole‐slide images were acquired on a Hamamatsu NanoZoomer slide scanner (model C12000‐02, NDP.scan version 3.4.0; Hamamatsu Photonics K. K., Hamamatsu, Japan) at 40× source magnification, yielding a pixel resolution of 0.227 μm/pixel (≈4.41 pixels/μm). Manual annotations made using QuPath open‐source software (version 0.3.2), https://qupath.github.io, last accessed 19 July 2026 [20].

### Data preparation and annotation

Training and validation data were derived from H&E‐stained liver sections from mice following 21 days of DDC diet. The primary training dataset consisted of a whole‐slide image (118,784 × 107,520 pixels) selected for abundant DR, with a focal region of blur excluded to prevent ambiguous training signals. To capture and account for heterogeneity in DR and staining characteristics, regions from sections from 50 more independent mice, each having undergone 21 days of DDC dietary injury as control animals in unrelated in‐house transgenic studies, were included. These sections were drawn from multiple staining batches and scanner runs and selected to represent diverse staining intensities, tissue morphologies, and inter‐animal variability in DR appearance at peak injury. All annotations were performed by a clinical pathologist, who annotated 1,596 polygon regions across training and validation images. Annotation followed a standardised protocol, with biliary populations identified based on morphological characteristics [21]. For model testing, 10 randomly sampled regions (each measuring 21,216 × 20,840 pixels) were selected from a further 10 unique 21‐day DDC diet animals not included in training or validation, with sections representing high DR prevalence.

### Model architecture, validation, and testing

Full details on model architecture and training are provided in the Supplementary materials and methods. Code for the DUCK‐Net model and analysis have been made publicly available at: https://github.com/WhatsMyPurpose/ducknet-dr-segmentation. Model validation was performed at the end of each training epoch using full‐image inference on the validation set. Final model performance was evaluated on the held‐out test set of 10 annotated samples, which were not used during training or hyperparameter selection. Final statistics for annotated pixels and tissue coverage in the training, validation, and test datasets are reported in the supplementary material, Table S1.

Four comparator architectures (U‐Net, U‐Net++, DeepLabv3+, and MA‐Net) were implemented using the segmentation_models_pytorch library with a ResNet34 encoder pretrained on ImageNet and were trained on the same training set as DUCK‐Net using matched hyperparameters (loss function, optimiser, learning rate, batch size, augmentation strategy, and number of epochs) [22, 23, 24, 25, 26]. For cross‐model evaluation in the CDE and MCD models, Dice coefficients were calculated against annotated DR regions, with DR burden calculated for each section as the annotated DR area divided by the total annotated tissue area, expressed as a percentage.

### Datasets

Raw spatial transcriptomic data (Stereo‐seq and scRNA‐seq) from Wu *et al* [12] were downloaded from the CNGB Nucleotide Sequence Archive (accession number CNP0003447; https://db.cngb.org/search/?q=CNP0003447). Processed H5AD files containing spatial gene expression profiles were accessed through the Cholestatic Injury and Repair Spatio‐Temporal Atlas (CIRSTA) database (https://db.cngb.org/stomics/datasets/STDS0000239/summary). Liver progenitor‐like cells (LPLC) and cholangiocyte (Chol) domain scores representing the spatial transcriptomic signatures of DRs were extracted for each timepoint (Days 0, 8, and 17 during DDC injury; Days 19, 24, and 38 during recovery) using the Python *anndata* library (version 0.9.2 https://anndata.readthedocs.io; last accessed 19 July 2026) called from R via the *R reticulate* package (version 1.28; https://cran.r-project.org/package=reticulate; last accessed 19 July 2026).

### Statistical analyses

Performance of the DUCK‐Net model against ground truth clinical annotations was evaluated via multiple metrics, including Dice coefficient, volumetric overlap, precision, recall (sensitivity), specificity, accuracy, and area under the receiver operating characteristic (ROC) curve. Unless otherwise stated, performance metrics are reported as mean ± SD. Segmentation across CNN architectures was compared using a non‐parametric Friedman test, given the paired design (each architecture evaluated on the same 10 test slides). Effect size was reported as Kendall's coefficient of concordance (W). To determine whether DUCK‐Net segmentation could be explained by individual IHC markers over the DDC time course, a multiple linear regression model was selected using DUCK‐Net coverage as the dependent variable and individual IHC markers of DR components as the independent variables (KRT19, SOX9, EpCAM, α‐SMA, PSR). Model fit was assessed using *R* ^2^ and *F* statistics. ROC analysis was performed to evaluate the discriminatory ability of DUCK‐Net and conventional IHC markers for classifying injury states in the DDC model (Supplementary materials and methods).

To enable comparison between published spatial transcriptomic data and our DUCK‐Net DDC time course (Days 0, 7, 14, and 21 injury; Days 28, and 35 recovery), both the Wu *et al* dataset and our DDC time course were interpolated to common timepoints (0, 7, 14, 21, 28, 35 days) using linear interpolation (approx() function in R, rule = 2). Interpolation was validated by comparison with cubic spline and LOESS methods. To account for different experimental protocols (21‐day versus 17‐day DDC treatment), time courses were normalised to percentage of protocol duration to demonstrate universal kinetics independent of absolute study duration. Linear regression was performed separately for injury and recovery phases to compare kinetic slopes. Pearson correlation coefficients were calculated between LPLC domain scores and each marker (DUCK‐Net, KRT19, EpCAM, SOX9, α‐SMA, PSR) using interpolated time courses. Statistical significance was assessed using the cor.test() function with two‐tailed tests.

## Results

To evaluate the ability of DUCK‐Net to quantify DRs from H&E morphology, we first assessed model performance against expert pathologist annotations on an independent test set of 10 diverse mouse liver sections selected to represent varying DR severities and tissue morphologies following 21 days of DDC dietary injury. Visual comparison demonstrated apparent close spatial concordance between DUCK‐Net predictions and pathologist‐annotated DR regions on both the initial training slide (Figure 1A,B) and the test set (Figure 1C), including accurate delineation around regions of small and large ducts, anastomosing ductular networks, and interface regions between portal tracts and parenchyma (Figure 1C). Quantitative evaluation across the test set demonstrated robust performance (supplementary material, Table S2), achieving a Dice coefficient of 0.85 ± 0.05 (Jaccard index 0.74), with consistency across diverse tissue appearances and staining variations (Figure 1D). Training curves demonstrated effective learning of the model, with validation loss decreasing rapidly initially before plateauing, indicating successful model convergence without overfitting over 88 epochs of training (Figure 1E). The model achieved robust performance metrics across the test set (Figure 1F; individual sample data is provided in the supplementary material, Table S2): precision 0.84 ± 0.07, recall 0.88 ± 0.08, mean pixel accuracy (MPA) 0.96 ± 0.01, and, notably, specificity was high (0.98 ± 0.01), indicating excellent discrimination against false positive detection.

**Figure 1:**
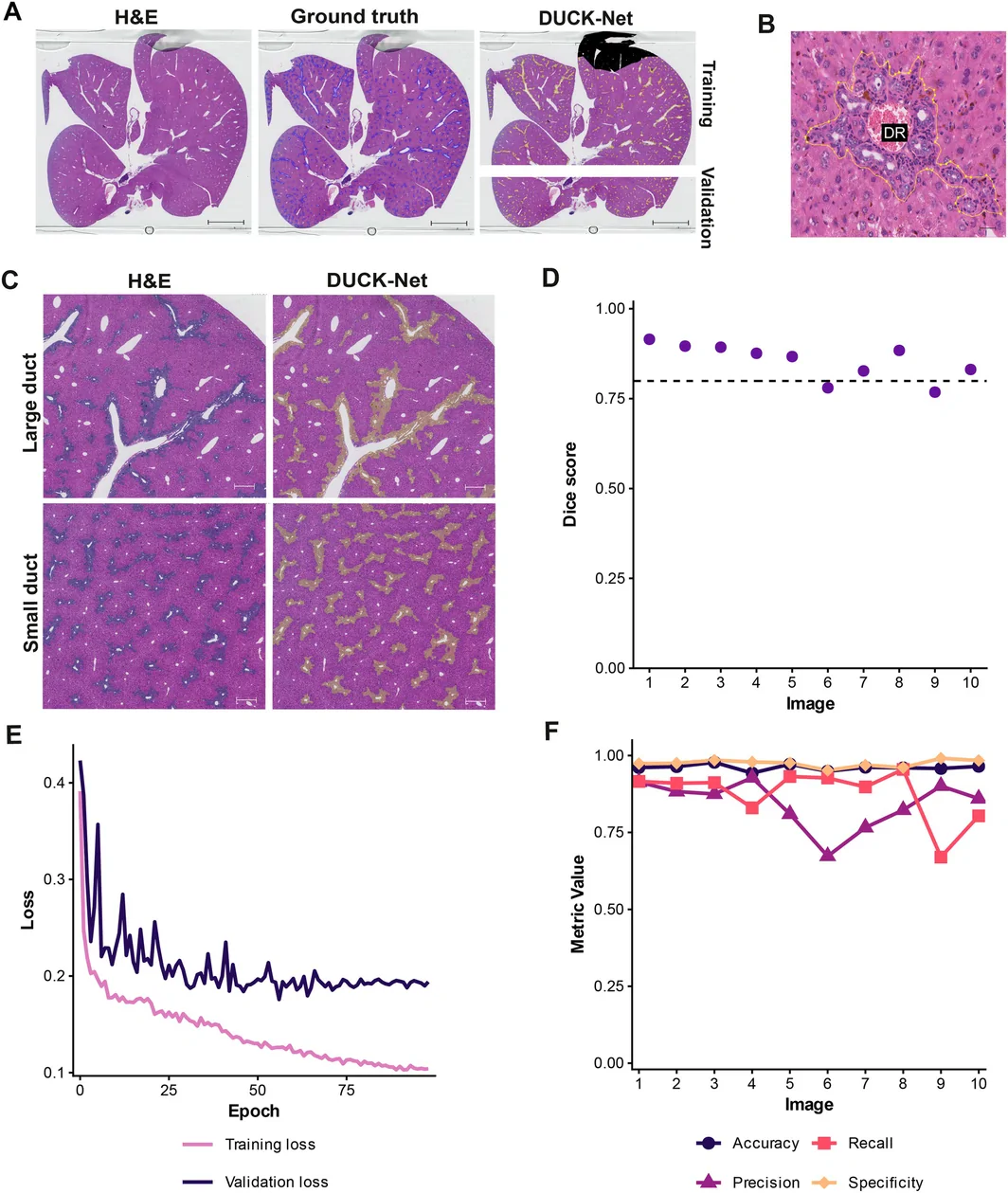
DUCK‐Net accurately quantifies DR from H&E morphology in mouse liver tissue following DDC dietary injury. (A) Representative mouse whole liver section (H&E) following 21 days of DDC treatment with expert pathologist annotations (ground truth) (blue overlay) and DUCK‐net segmentation (yellow overlay) in training and validation regions of initial training slide. Artefact masking and eosin channel enhancement within training dataset. Scale bars, 5 mm. (B) High‐magnification view of DR region as annotated (yellow) by clinical liver histopathologist, capturing epithelial ductular structures and surrounding reactive stroma. Scale bar, 100 μm. (C) Representative H&E and DUCK‐Net predictions from test set showing segmentation across diverse morphologies including large ducts (top) and small ducts (bottom) and interface regions. Scale bars, 200 μm. (D) Dice coefficients for 10 test samples (mean 0.85 ± 0.05) demonstrating consistent performance across varying DR severities. Dashed line indicates mean value. (E) Training and validation loss curves across 88 epochs. Smooth convergence indicates effective learning without overfitting. (F) DUCK‐Net performance across 10 test samples including precision, recall (sensitivity), specificity, and accuracy.

To contextualise the performance of DUCK‐Net for DR detection, we benchmarked its performance against four established CNN‐based segmentation architectures: U‐Net, U‐Net++, DeepLabv3+, and MA‐Net [23, 24, 25, 26]. All five architectures achieved closely comparable performance on the test datasets, with Dice coefficients ranging from 0.851 to 0.857 (DUCK‐Net 0.854 ± 0.053) (supplementary material, Table S3, supplementary material, Figure S1). A Friedman test detected no significant difference in Dice scores across architectures [*χ* ^2^ (4) = 7.76, *p* = 0.101; Kendall's *W* = 0.194]. The inter‐architecture range (0.006) was substantially smaller than the between‐slide variability (0.053). Similar findings were obtained on the validation dataset, where the best Dice scores ranged from 0.809 (DeepLabv3+) to 0.824 (MA‐Net), with DUCK‐Net at 0.821.

To assess whether DUCK‐Net segmentation was able to faithfully map the appearance, progression, and resolution of DRs, DUCK‐Net coverage was quantified on H&E‐stained sections from a DDC‐induced model of liver injury and recovery (0.1% DDC diet, *n* = 4 per timepoint) (Figure 2A). A panel of core established IHC markers was selected to characterise the spatiotemporal dynamics of DRs: KRT19, SOX9, and EpCAM for ductular epithelium and α‐SMA and PSR for the associated stromal response (Figure 2B). DUCK‐Net coverage increased progressively during DDC injury, peaking at Day 21 before declining during the recovery phase (Figure 2C). This temporal pattern closely mirrored the dynamics of all five IHC markers. Notably, DUCK‐Net detected the onset of ductular reaction at Day 7 and captured the expected lag in resolution during recovery, consistent with the known biology of DR regression.

**Figure 2:**
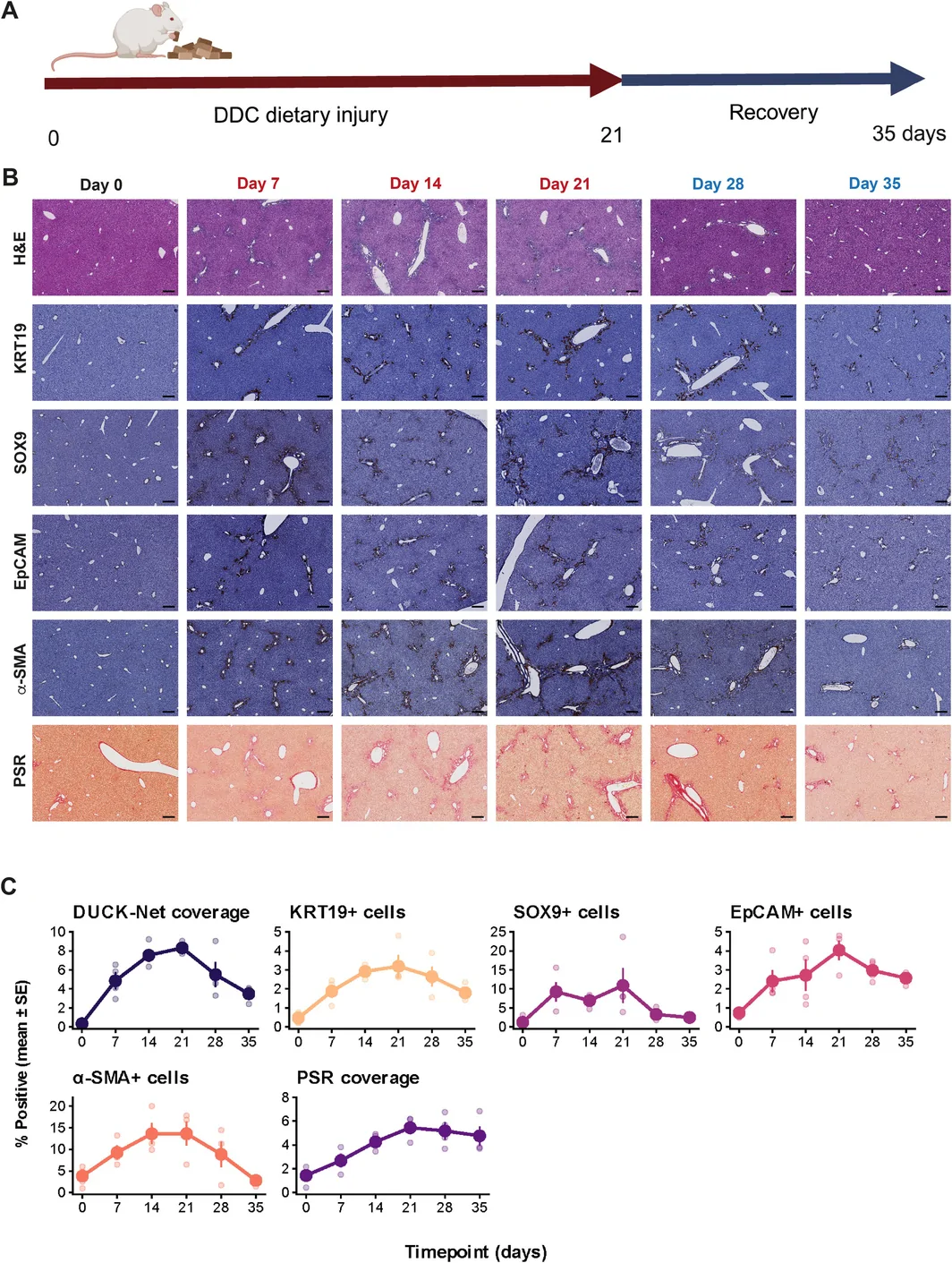
DUCK‐Net and IHC marker quantification across DDC‐induced liver injury and recovery. (A) Schematic of experimental timeline. Mice were fed 0.1% DDC diet for up to 21 days followed by 14 days of recovery on normal chow (*n* = 4 per timepoint). Figure created in BioRender. Guest, R. (2026) https://BioRender.com/bxexlm6. (B) Representative images of liver sections stained with H&E or IHC staining of markers of DR across the DDC time course: KRT19, SOX9, EpCAM, α‐SMA, and PSR. Scale bars, 200 μm. (C) Quantification of DUCK‐Net coverage and IHC markers across the time course. DUCK‐Net coverage was expressed as percentage coverage on H&E sections. IHC markers were quantified as percentage positive cells (KRT19, SOX9, EpCAM, α‐SMA) or percentage area (PSR). Markers show a progressive increase during injury (Days 0–21) and decline during recovery (Days 28–35). Data are shown as mean ± SE (*n* = 4 per timepoint).

To investigate whether DUCK‐Net coverage is able to fully capture the DR niche and representation of both epithelia and stroma, a multiple linear regression model was constructed to model DUCK‐Net variance as a function of core DR components (KRT19, SOX9, EpCAM, α‐SMA, PSR) across the DDC time course (Table 1). Strong correlations were observed between DUCK‐Net coverage and all IHC markers individually (KRT19 *r* = 0.90, *p* < 0.0001; α‐SMA *r* = 0.69, *p* = 0.0005; EpCAM *r* = 0.55, *p* = 0.0100; PSR *r* = 0.52, *p* = 0.015; and SOX9 *r* = 0.51, *p* = 0.019) (Figure 3A). Model assumptions were validated by examining pairwise relationships between all variables using scatterplot matrices and residual diagnostics (supplementary material, Figures S2 and S3). Strong agreement was observed between IHC‐predicted and actual DUCK‐Net values across individual samples from all timepoints (*R* ^2^ = 0.88, RMSE = 0.89) (Figure 3B), and DUCK‐Net closely tracked predicted values throughout injury and recovery, with the model explaining a high proportion of the variance in DUCK‐Net coverage across the time course (*R* ^2^ = 0.88, *F*_5,15_ = 22.90, *p* = 1.58 × 10^−6^) (Figure 3C). Analysis of individual marker contributions revealed that KRT19 was the dominant predictor (standardised *β* = 0.65, *p* = 2.8 × 10^−4^), with SOX9 also contributing significantly (standardised *β* = 0.26, *p* = 0.0444). The stromal markers α‐SMA and PSR did not reach significance as independent predictors in the multivariate model (Figure 3D). Despite the observed moderate correlation between KRT19 and SOX9 (*r =* 0.33, *p =* 0.15), both epithelial markers remained independent predictors, suggesting they capture at least partially distinct populations within the ductular epithelium (Figure 3E). EpCAM, which marks ductular epithelial cells alongside a broader population of progenitor‐like cells, showed moderate correlation with KRT19 (*r* = 0.52, *p* < 0.05) and weak, non‐significant correlation with SOX9 (*r* = 0.30) (Figure 3A) and did not contribute independent predictive variance to the model (standardised *β* = 0.02, *p* = 0.86; Figure 3D). EpCAM's predictive content was therefore largely redundant with KRT19, consistent with the two markers labelling overlapping populations of ductular epithelial cells; KRT19 alone is sufficient to capture the EpCAM‐relevant variation in DUCK‐Net coverage. Twelve per cent of DUCK‐Net variance was not explained by the model and likely reflected additional morphological features not captured by this core IHC panel such as inflammatory infiltrate, tissue architecture, or cellular morphology. This was maximal at the point of peak injury (Days 14–21), when DUCK‐Net tended to exceed predicted values (Figure 3F).

**Table 1:** Multiple linear regression coefficients (DUCK‐Net versus IHC markers).

Predictor	Beta	SE	Standardised *β*	CI 95 lower	CI 95 upper	*T* value	*p* value
(Intercept)	−2.09	0.84	‐	−3.88	−0.31	−2.50	0.0243
KRT19	2.13	0.45	0.65	1.16	3.09	4.70	< 0.001
α‐SMA	0.05	0.06	0.11	−0.07	0.18	0.89	0.3875
PSR	0.25	0.22	0.15	−0.22	0.72	1.14	0.2706
SOX9	0.20	0.09	0.26	0.01	0.39	2.19	0.0444
EpCAM	0.05	0.26	0.02	−0.51	0.61	0.18	0.8580

*R* ^2^ = 0.88, adjusted *R* ^2^ = 0.85, *F*_5,15_ = 22.90, *p* = 1.58e‐06.

Model fit statistics from multiple linear regression analysis modelling DUCK‐Net coverage as a function of core DR components across DDC injury and recovery time course. Predictor variables comprised epithelial markers (KRT19, SOX9, EpCAM) and stromal markers (α‐SMA, PSR). Beta, unstandardised regression coefficient; SE, standard error; standardised *β*, regression coefficient normalised to allow comparison of relative predictor contributions; CI 95% CI for unstandardised *β*. KRT19 and SOX9 were significant independent predictors of DUCK‐Net coverage.

**Figure 3:**
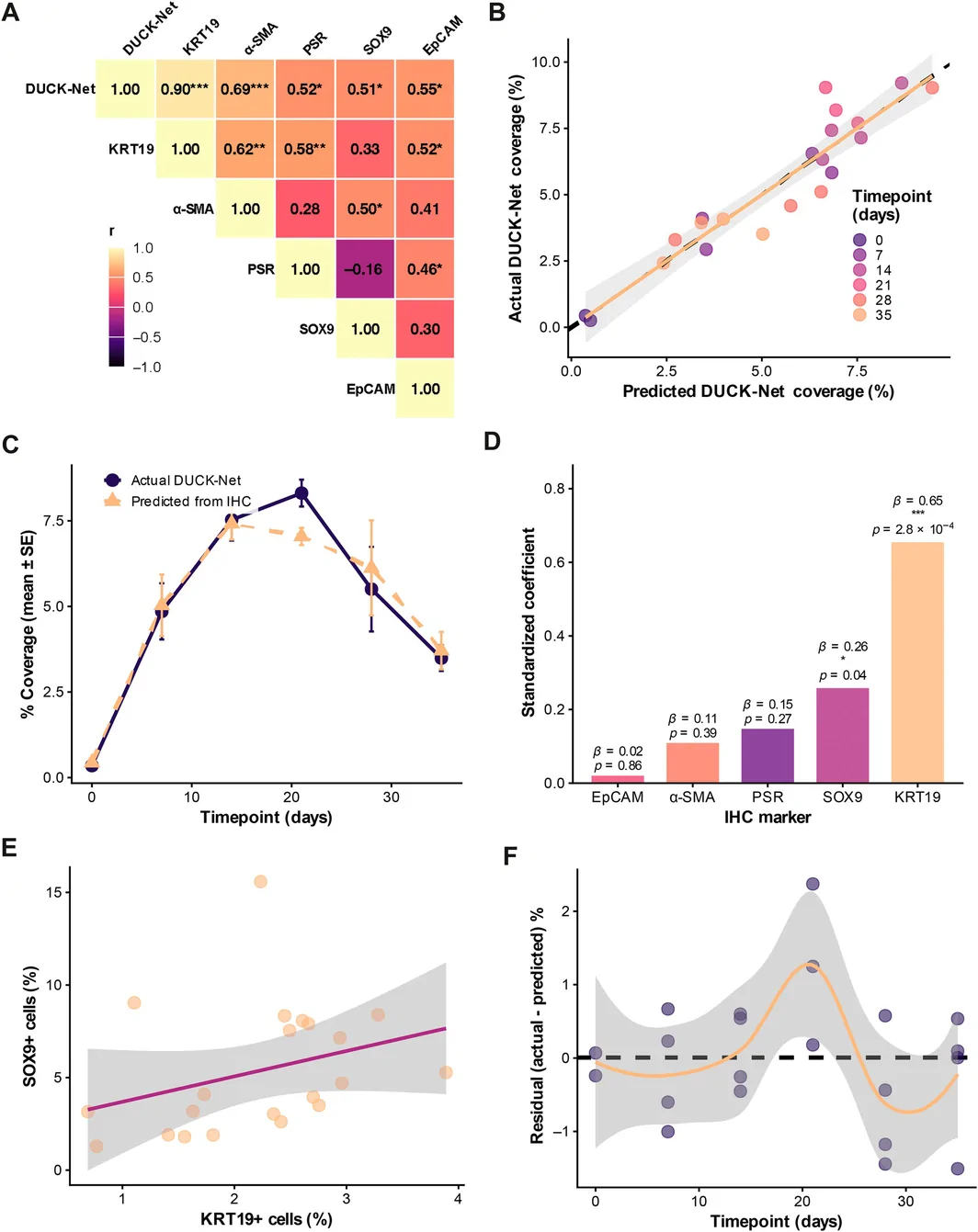
DUCK‐Net captures DR niche as characterised by IHC markers across a time course of DDC injury and recovery. (A) Correlation matrix showing Pearson correlation coefficients between DUCK‐Net coverage and individual IHC markers (KRT19, α‐SMA, PSR, SOX9, EpCAM). Colour intensity reflects correlation strength (colour scale, left; *r* = −1 to 1). Significance levels: *p < 0.05, **p < 0.01, ***p < 0.001. (B) Scatter plot of predicted versus actual DUCK‐Net coverage for individual samples. Each point represents one sample, coloured by timepoint (days). Dashed line indicates perfect agreement (*y* = *x*); orange line shows linear regression fit with 95% CI. *R* ^2^ = 0.88, RMSE = 0.89. (C) DUCK‐Net coverage (actual, blue) versus IHC predicted values (orange, dashed) across the DDC diet injury and recovery time course. Data shown as mean ± SE (*n* = 4 per timepoint). Model: *R* ^2^ = 0.88, *F*_5,15_ = 22.90, *p* = 1.58 × 10^−6^. (D) Standardised regression coefficients showing relative contribution of each IHC marker to DUCK‐Net prediction. Significance levels: ns not significant, *p < 0.05, ***p < 0.001. (E) Scatter plot of SOX9 versus KRT19 expression, demonstrating moderate correlation between epithelial markers (*r* = 0.33, *p* = 0.15). Line shows linear regression fit with 95% CI. (F) Model residuals (actual − predicted DUCK‐Net coverage) across the time course. Dashed line indicates zero residual; orange line shows LOESS smoothed trend with 95% CI. Positive residuals at Days 14–21 indicate DUCK‐Net exceeds predicted values at peak injury.

To evaluate the discriminatory performance of DUCK‐Net relative to conventional IHC markers, ROC analysis was performed, assigning clinically relevant categories (uninjured liver Day 0; any injury Days 7–35; peak injury Days 14–21; early injury Day 0–7 and recovery Days 28–35) (Figure 4; supplementary material, Table S4). For distinguishing control from injured liver, DUCK‐Net achieved perfect discrimination (AUC = 1.00), equivalent to KRT19 (AUC = 1.00) and outperforming SOX9 (AUC = 0.87). Similarly, for identifying peak versus early injury, DUCK‐Net demonstrated excellent performance [AUC = 0.98, comparable to KRT19 (AUC = 1.00) and PSR (AUC = 0.98)], while SOX9 showed no discriminatory ability (AUC = 0.50). Interestingly, in distinguishing recovery from any injury, SOX9 demonstrated superior discrimination (AUC = 0.97) followed by α‐SMA (AUC = 0.82) and DUCK‐Net (AUC = 0.81), while KRT19 and PSR performed poorly (AUC = 0.66 for both), reflecting the distinct biological processes captured by each marker.

**Figure 4:**
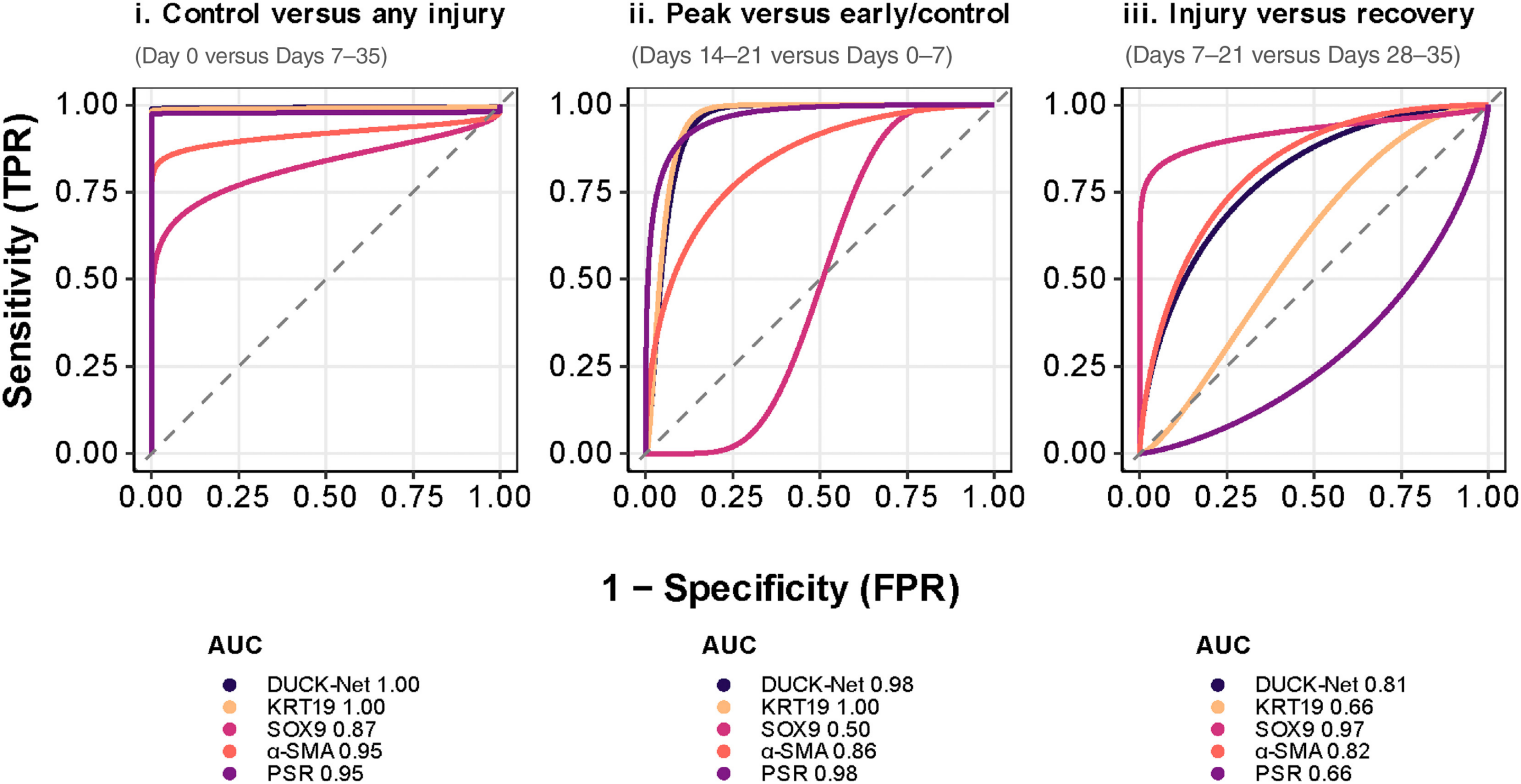
Discriminatory performance of DUCK‐Net in identification of liver injury and recovery. ROC curves comparing discriminatory performance of DUCK‐Net (blue) with conventional IHC markers (KRT19, SOX9, α‐SMA, and PSR). (i) Control versus any injury (Day 0 versus Days 7–35). (ii) Peak injury versus early/control timepoints (Days 14–21 versus Days 0–7). (iii) Active injury versus recovery phase (Days 7–21 versus Days 28–35). AUC values are shown below individual plots. Dashed diagonal line indicates performance of a random classifier (AUC = 0.50). Curves were smoothed using binormal fitting for visualisation only; all AUC values were calculated from empirical data. *n* = 4 per timepoint.

To test the limits of DUCK‐Net beyond its training context, we applied the model without re‐training to two mechanistically distinct models of liver injury that exhibit DRs: the CDE diet and the MCD diet. Dice coefficients were lower than those achieved on the DDC model (CDE Dice 0.41 ± 0.07; MCD 0.33 ± 0.19) and tended to scale with DR burden (supplementary material, Figure S4).

Several comprehensive molecular characterisations of the DR niche using single‐cell resolution techniques have been described, including DDC‐induced cholestatic liver injury [27, 28]. These have included studies using spatially enhanced resolution omics sequencing (Stereo‐seq), a DNA nanoball method that applies high‐resolution gene expression data to a wide spatial area, ensuring signals from rare cell populations are not diluted by surrounding common cell types and allowing characterisation of regional variations across tissues [12]. To investigate whether DUCK‐Net has potential to capture the molecular dynamics of the DDC damage response, publicly available raw spatial transcriptomic (Stereo‐seq) and single‐cell RNA‐seq data as published by Wu *et al* [12] were accessed from the CNGB Nucleotide Sequence Archive through the CIRSTA interactive database. Time course alignment was conducted using linear interpolation and normalised to protocol duration. The proportions of Chol (*Krt19* ^*+*^, *Krt7* ^*+*^) and LPLC (*Spp1* ^*+*^, *Sox9* ^*+*^, *Krt19* ^*−*^, *Krt7* ^*−*^) spatial domains were extracted at each timepoint and compared with DUCK‐Net coverage (supplementary material, Table S5). DUCK‐Net and LPLC domains shared similar kinetic trajectories during injury and recovery phases, both peaking at the point of DDC withdrawal and exhibiting progressive recovery but incomplete resolution at the end of the model (Figure 5A). In contrast, the Chol domain showed little dynamic change throughout the time course, with approximately nine‐fold smaller magnitude of response compared to LPLC.

**Figure 5:**
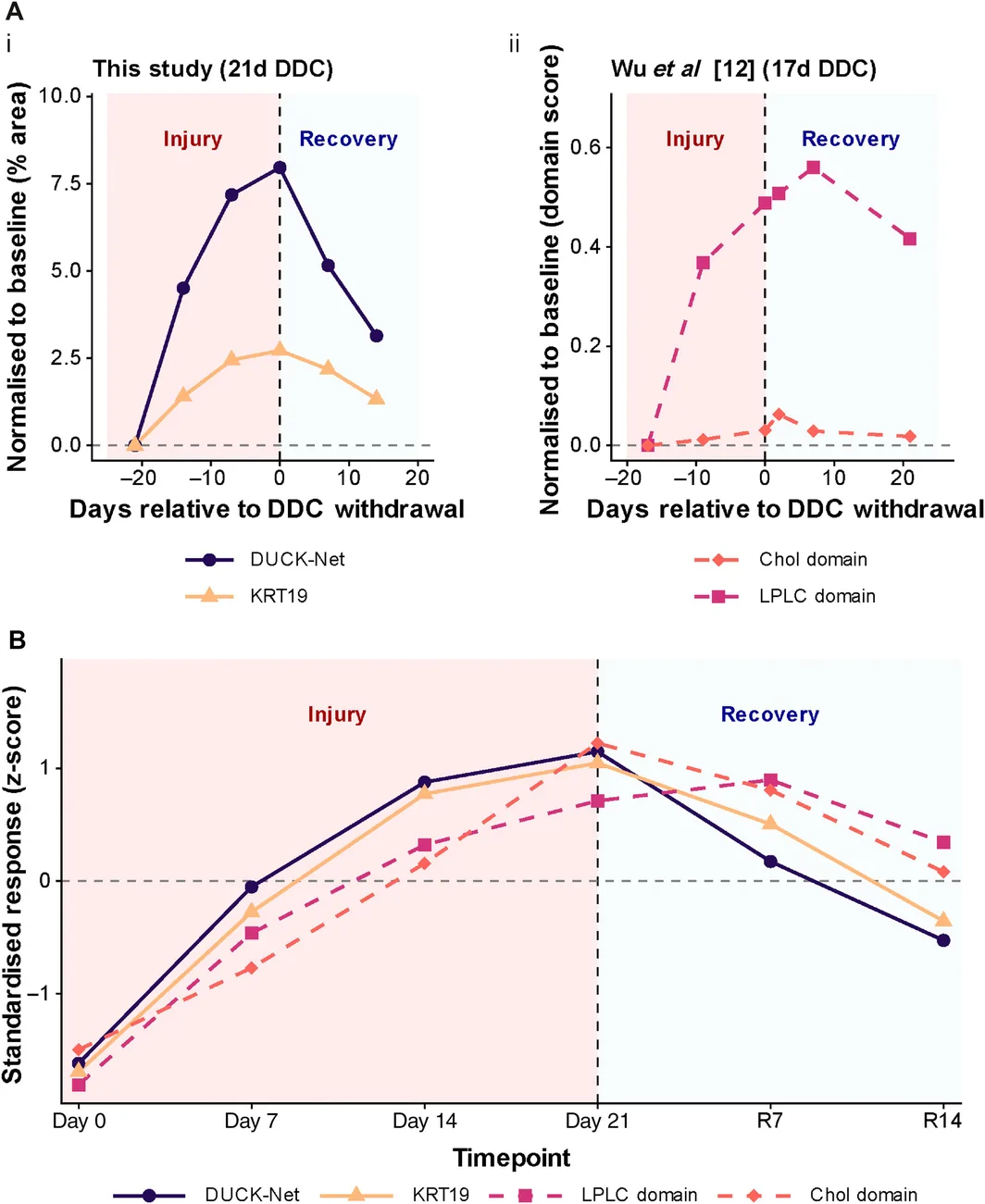
DUCK‐Net correlates with spatial transcriptomics‐defined liver progenitor cell domains from an independent dataset. (A) Universal DR kinetics across the two studies. DUCK‐Net coverage and KRT19 quantification were normalised to protocol duration and compared with LPLC and Chol domain coverage from spatial transcriptomic (Stereo‐seq) data as published by Wu *et al* [12] accessed from the CNGB Nucleotide Sequence Archive. Timepoints were expressed as days relative to withdrawal of the DDC diet. Values were normalised to baseline expression within each dataset. Linear interpolation was applied to estimate values at equivalent timepoints between studies. Datasets show concordant kinetics between DUCK‐Net and LPLC domains, with progressive increase during injury and decline during recovery, demonstrating DR dynamics are conserved across experimental models and quantification methods. Shaded regions indicate injury phase (pale red) and recovery phase (pale blue). (B) Standardised (z‐normalised) temporal trajectories of DUCK‐Net coverage and KRT19 immunohistochemistry (this study; solid lines) and of LPLC and Chol spatial‐domain scores from the CIRSTA atlas (Wu *et al* [12]; dashed lines) across the DDC injury‐recovery time course. Shaded regions indicate injury phase (pale red) and recovery phase (pale blue).

To quantify trajectory similarity while accounting for differences in response magnitude, dynamic time warping (DTW) was selected to enable quantitative comparison of trajectories in the two models. DTW measures the similarity between two temporal sequences by computing the minimum cumulative distance required to establish the optimal alignment between them and yields an output distance measure where lower values indicate more similar trajectories [29, 30, 31]. Z‐normalisation of each trajectory (centred on its mean and scaled to unit standard deviation) allows the temporal shape of each curve to be compared independently of absolute magnitude [32]. This analysis revealed that DUCK‐Net trajectories followed similar rise‐peak‐resolution kinetics to KRT19 IHC as well as the LPLC and Chol spatial domains (Figure 5B). DUCK‐Net most closely matched KRT19 (DTW distance 0.16) but was comparably distant from the LPLC (0.27) and Chol domains (0.27) (Table 2).

**Table 2:** Dynamic time warping analysis of DUCK‐Net and CIRSTA spatial domains.

Comparison	DTW distance	DTW similarity
DUCK‐Net versus LPLC	0.266	0.790
DUCK‐Net versus Chol	0.273	0.786
DUCK‐Net versus KRT19	0.161	0.861
KRT19 versus LPLC	0.209	0.827
KRT19 versus Chol	0.329	0.752
LPLC versus Chol	0.246	0.803

Pairwise DTW analysis comparing temporal trajectories of DUCK‐Net coverage and IHC marker (KRT19) quantification over DDC injury/recovery time course with LPLC and Chol spatial domain proportions from published Stereo‐seq data (Wu *et al*, *Nature Genetics* 2024). DTW distance represents minimum cumulative distance required for optimal alignment between two temporal sequences; lower values indicate greater trajectory similarity. Prior to analysis, each trajectory was z‐normalised (centred on its mean and scaled to unit standard deviation). DTW similarity was calculated as 1/(1 + DTW distance). All time courses were normalised to baseline (Day 0) values. DUCK‐Net trajectories most closely matched KRT19 (DTW distance 0.16) and were comparably aligned with the LPLC and Chol spatial domains (DTW distance 0.27 for both).

## Discussion

The development of deep learning algorithms to accurately and rapidly analyse medical images has generated considerable interest in the diagnosis, management, and understanding of an array of disease states [33]. The application of these tools to H&E‐stained tissues has opened up new possibilities for the prediction of molecular properties from morphological features, such as microsatellite instability and even patterns beyond human visual perception [34]. Computer vision, specifically CNN architectures, have been widely adopted as the models of choice for digital image segmentation, in particular analysis of digital histopathology and H&E‐stained whole slide images [35]. These have included architectures capable of creating highly detailed segmentation maps using limited training samples with extremely fast learning capability [36]. DUCK‐Net builds on such models using a convolutional block to improve accuracy of prediction of complex or irregular border locations and residual down‐sampling to utilise image information at each resolution level, keeping the original field of view alongside the processed input image. This prevents loss of multi‐scale information to improve small target detection [16].

We hypothesised that the architectural features of DUCK‐Net would make it well suited to detect DRs in mouse liver injury models. Although multi‐scale feature aggregation can be achieved by alternative CNN architectures using mechanisms such as Atrous Spatial Pyramid Pooling (ASPP) in DeepLabv3+ or dense nested skip pathways in U‐Net++, we show DUCK‐Net performs competitively while retaining the ability to resolve features across the range of scales at which DRs are present. DRs frequently do not exhibit sharp, well‐defined boundaries and the interface regions between portal tracts, and the parenchyma can be complex and irregular. Furthermore, DRs contain heterogenous histological structures which simultaneously span multiple scales: small individual cells including HPCs and ductules, large anastomosing ductular networks as well as the surrounding stroma, and ECM all exhibit different H&E staining characteristics. Given the well‐documented spectrum of histopathological phenotypes across disease pathologies, various published classification systems, and challenges in annotation, DRs would appear to be a well‐suited application of a standardised automated detection method easily and accurately trained on a small training cohort [21, 37, 38].

The Dice similarity coefficient of 0.85 ± 0.05 achieved in our test set (ranging from 0, indicating no spatial overlap between two binary segmentation results, to 1, indicating complete overlap) would be generally categorised as ‘good to very good accuracy’, although some consideration of task complexity should be accounted for. Segmentation of a well‐defined structure (such as solid organ segmentation in CT, for example in liver volumetry or radiotherapy planning) will likely achieve a higher Dice score than segmentation of complex irregular structures with poorly defined boundaries. The range of Dice scores across the test set (0.77–0.91) likely reflects differences in DR severity, morphological complexity, or staining characteristics. Although few published studies directly measured inter‐observer variability of pathologist annotation specifically of DR regions, related liver histopathology assessments typically have showed only moderate, at best, agreement (weighted kappa values 0.5–0.7), highlighting the challenge of subjective histological assessment and the potential of standardised automated detection [39, 40, 41]. The close tracking between training and validation loss throughout training confirms the model generalises well beyond the training data, and the high specificity achieved (0.98) indicates excellent discrimination against false detection – a crucial factor in histopathological analysis. The robust segmentation performance achieved from 51 training slides contrasts sharply with many deep learning applications which require hundreds of thousands of annotated images and offers considerable opportunities for research workflows in particular, where expert pathologist annotation time can represent a significant bottleneck. The precision (0.84) and recall (0.88) of the model were well balanced, indicating that the network is neither over‐ nor under‐segmenting DR regions.

The strong correlation between DUCK‐Net coverage and KRT19+ cells (*r* = 0.90), together with the wide dynamic range observed across the DDC time course, establish DUCK‐Net as a continuous quantitative measure of DR severity comparable to established IHC‐based quantification. Regression analyses revealed KRT19 as the dominant predictor of DUCK‐Net coverage (*β* = 2.13, *p* < 0.001), with SOX9 contributing additional independent variance in response to liver injury (*β* = 0.20, *p* = 0.044). The inability of α‐SMA and PSR to reach significance as independent predictors despite moderate individual correlations suggests the stromal response is largely covariant with epithelial expansion, rather than contributing unique predictive information – reinforcing that DUCK‐Net is primarily capturing changes driven by epithelial morphology [42, 43]. Using an IHC panel intentionally restricted to five core epithelial and stromal markers, the model explained 88% of DUCK‐Net variance, with residual unexplained variance (12%) maximal between days 14 and 21, when DUCK‐Net exceeded IHC‐predicted values. This timing coincides with peak inflammatory infiltration in the DR niche, when ductular proliferation is accompanied by dense macrophage and neutrophil influx serving to promote and sustain epithelial expansion [44, 45]. This aligns with data from Wu *et al*, who demonstrated that lipid‐associated macrophage (LAM)‐Chol interactions peak at maximal injury. H&E segmentation using DUCK‐Net therefore offers potential to capture additional biological information not covered by this panel and, unlike IHC, which provides binary protein detection, offers continuous morphological information such as cellular architecture, nuclear features, and tissue organisation. Such potential advantages of deep‐learning‐based H&E analysis were further illustrated by ROC analysis, which showed that DUCK‐Net, unlike any single IHC marker, demonstrated excellent discrimination across multiple clinically relevant comparisons: control versus injury (AUC 0.98, comparable to KRT19) and injury versus recovery (AUC 0.81).

The strong alignment between DUCK‐Net and the spatial transcriptomic domains described by Wu *et al* [12], despite differences in experimental design, mouse strain, and quantification methods, indicates that the kinetics of the DR in the DDC injury/recovery model are conserved and that H&E‐based deep learning can recapitulate the temporal dynamics of the DR niche at the molecular level. Both conventional Pearson correlation and z‐normalised DTW indicated that DUCK‐Net coverage was able to track both the LPLC and Chol domains, consistent with lineage tracing studies showing that DRs arise through activation and expansion of HPCs that subsequently develop into reactive cholangiocytes [27, 46]. DUCK‐Net therefore appears to capture the ductular reaction as an integrated morphological entity.

Considered together, these analyses clarify the comparative value of a H&E‐based segmentation approach over single‐marker IHC quantification. Conceptually, no single marker – including KRT19 – fully accounts for DUCK‐Net coverage, indicating that the model captures DR as an integrated multicellular niche rather than a single epithelial compartment, consistent with its concordance with the multicellular LPLC/Chol domains. Practically, the substantial benefits of the H&E‐based approach are evident, one of the most important arguably being its retrospective applicability to archival or digitised cohorts in which tissue is depleted. The present single‐class model quantifies the spatial extent of the DR rather than resolving its individual cellular, stromal, vascular, and inflammatory components, the integration of which represents a natural extension of this work.

An important consideration is that in this study, DUCK‐Net was tested and validated exclusively using the DDC dietary model of biliary injury. While DDC produces a reliable and robust DR with characteristic periportal expansion, caution is warranted in extrapolating these findings to other contexts such as the centrilobular reactions seen in hypoxic injury or extensive HPC activation characteristic of massive hepatic necrosis. The direct application of DUCK‐Net to other settings of liver injury without re‐training serves to illustrate how strongly the surrounding histological context of the training set governs the ultimate performance of the model. Not only is accuracy driven by the cellular morphology and staining characteristics of the target feature (in this instance the DR region itself), but the surrounding structures and their interface to the DR are also of critical importance. For example, the MCD diet is a widely used model of steatohepatitis featuring DRs, usually at a lower frequency than the CDE or DDC models. The histology of MCD is characterised by hepatocytes distended with macrovesicular lipid alongside ballooning degeneration and infiltrating inflammatory cells which immediately surround DRs. Presented with this novel DR‐parenchymal interface, the model as currently trained is unable to recognise DRs in this unfamiliar context and detection accuracy falls.

Further to this, a central translational potential for DUCK‐Net is its applicability to human liver disease. DRs are a feature of a diverse range of liver pathologies, including PBC, PSC, MASLD, and biliary atresia, where distinct DR phenotypes are observed defined by their unique aetiologies. While DUCK‐Net was in part selected for its accessibility and deployment on standard research hardware, robust deployment of the model to diverse contexts such as these will require disease‐ and feature‐specific re‐training using expert‐annotated human material and validation across cohorts. The greater morphological heterogeneity of human DRs or indeed any other histopathological feature across aetiologies may render this translational setting particularly amenable to histology foundation models, whose large‐scale pretraining is designed to transfer across diverse tissue contexts. Such approaches represent a complementary avenue to disease‐specific re‐training of this CNN and an important direction for future work.

The potential of DUCK‐Net to offer enhanced discriminatory potential over classic IHC markers to capture the spatiotemporal dynamics of liver injury and recovery, better reflecting the molecular response of the ductular reaction, makes this a promising accessible research tool with few computational costs. Although our test set was selected to reflect a spectrum of staining intensities, DDC response, and image artefacts and performed well during external validation, DUCK‐Net can be re‐trained on institution‐specific data to optimise colour normalisation and generalisability. Beyond accuracy, the practical advantages for research workflows are considerable: rapid and standardised quantification of DRs otherwise requiring serial sectioning for IHC panel and/or specialist pathologist annotation. Realisation of its full potential will require systematic evaluation across the diverse liver pathologies and clinical contexts in which DRs are observed.

## Author contributions statement

NF, KW, EMH, TJK and RVG were responsible for conceptualisation. NF, KW, HW, TYM, W‐YL, TJK and RVG developed the methodology. NF, KW, TJK and HW were responsible for software. NF, KW, DF, TJK and RVG performed validation. NF, KW, CM, DF and RVG carried out the formal analysis. NF, KW, DF, CM, KD and RVG conducted the investigation. LB, SJF, TYM and RVG provided resources. NF, KW, CM and RVG were responsible for data curation. NF and RVG wrote the original draft. NF, KW, ST, EMH, SJW, SJF, TJK and RVG reviewed and edited the manuscript. NF, KW and RVG were responsible for visualisation. IPT and RVG supervised the study. NF and RVG were responsible for project administration. IPT and RVG acquired funding. All authors approved the final version of the manuscript.

## Supporting information


Supplementary materials and methods
**Figure S1.** Benchmarking of DUCK‐Net against CNN segmentation architectures
**Figure S2.** Pairwise relationships between DUCK‐Net coverage and IHC markers across DDC time course
**Figure S3.** Regression model diagnostics for multiple linear regression of DUCK‐Net coverage against IHC markers
**Figure S4.** Cross‐model evaluation of DUCK‐Net in CDE and MCD models of liver injury
**Table S1.** Annotated pixel statistics and dataset composition for model development and evaluation
**Table S2.** DUCK‐Net performance metrics for individual test samples
**Table S3.** Benchmarking of DUCK‐Net against established CNN‐based segmentation architectures
**Table S4.** ROC analysis summary for DUCK‐Net and IHC markers across clinically relevant comparisons
**Table S5.** Time course quantification of DUCK‐Net, IHC markers, and interpolated spatial transcriptomic domains

## Data Availability

The DUCK‐Net model and analysis code are publicly available at https://github.com/WhatsMyPurpose/ducknet-dr-segmentation. Raw spatial transcriptomic data from Wu *et al* were accessed from the CNGB Nucleotide Sequence Archive (https://db.cngb.org/cnsa/ accession CNP0003447) and CIRSTA database (https://db.cngb.org/stomics/cirsta/).
