# Supplementary material for: DUCK‐Net: automated deep learning segmentation of ductular reactions in murine liver injury captures multicellular niche dynamics from H&E morphology
Source: J Pathol. 2026 Sep 8;270(4):441–54. doi: 10.1002/path.70113 (PMC13728815; doi:10.1002/path.70113)
Supplement: Supplementary file 1 [file PATH-270-441-s001.docx]

**DUCK-Net: automated deep learning segmentation of ductular reactions in murine liver injury captures multicellular niche dynamics from H&E morphology**

N Feeley *et al. J Pathol* <https://doi.org/10.1002/path.70113>

**Supplementary Figures S1–S4**

**Supplementary Tables S1–S5**

Reference numbers refer to the main text list.

**Supplementary materials and methods**

Whole-slide images (WSIs) in NDPI format were processed using OpenSlide (<https://openslide.org>**; last accessed 19 July 2026)** and down-sampled by a factor of 8, providing sufficient preservation of tissue microstructure while allowing practical patch dimensions for deep learning. Pathologist annotations, exported from QuPath (version 0.3.2; <https://qupath.github.io>; last accessed 19 July 2026) as JSON polygon objects, frequently encompassed vascular lumina; these were excluded through intensity thresholding followed by elliptical morphological operations via OpenCV [47]. All image processing was implemented in Python (version 3.11; <https://www.python.org>; last accessed 19 July 2026).

The DUCK-Net model was configured with 17 initial filters. The model was trained on randomly sampled RGB patches of 512 × 512 × 3 pixels. To address the class imbalance inherent in DR segmentation, a stratified sampling strategy was implemented: sampling probability for each source image was weighted proportionally to total tissue area, and at least 50% of patches per batch were constrained to contain DR tissue. Training proceeded with a batch size of 4 and 2,000 samples per epoch. To improve generalisation and mitigate overfitting, data augmentation was implemented using the Albumentations library [48]. Geometric augmentations including random rotations and flips were applied, along with Gaussian blur. Stain variation was addressed through a custom haematoxylin–eosin–DAB (HED) colour jitter augmentation, wherein images were transformed to HED colour space, the H&E channels were independently jittered, and the image was transformed back to RGB space.

Model optimisation employed the Dice coefficient as the objective function. Learning rate adjustment followed a reduce-on-plateau strategy, monitoring the validation Dice coefficient with a reduction factor of 0.5 and patience of 10 epochs. Training was conducted on an NVIDIA RTX 6000 Ada graphics processing unit (NVIDIA Corp., Santa Clara, CA, USA) with 48 GB memory, completing in 8 h over 88 epochs.

For validation of the model, a sliding window approach was employed with 512 × 512-pixel patches and 35% overlap between adjacent windows. Overlapping predictions were combined using a logical OR operation to produce full-resolution segmentation maps. The Dice coefficient was computed across the entire validation set, and model checkpoints were saved when validation Dice improved.

Area under the ROC curves (AUC) was calculated from empirical (non-parametric) ROC curves with 95% CIs estimated using the DeLong method [49]. Optimal classification thresholds were determined by maximising Youden’s J statistic (sensitivity + specificity − 1). Pairwise comparisons of AUC between DUCK-Net and each marker were performed using the DeLong test for correlated ROC curves. ROC curves were smoothed using binormal fitting for visualisation purposes only; this approach models the score distributions of each class as normal and derives a parametric ROC curve accordingly. All ROC analyses were performed in R using the pROC package (**version 2026.01.1+403; R Foundation for Statistical Computing, Vienna, Austria;** <https://www.r-project.org>**; last accessed 19 July 2026**) using the pROC package (**version 1.19.0.1;** <https://cran.r-project.org/package=pROC>**; last accessed 19 July 2026**).

**Figure S1. Benchmarking of DUCK-Net against CNN segmentation architectures.** Dice coefficients for DUCK-Net and four established CNN segmentation architectures (U-Net, U-Net++, DeepLabv3+, MA-Net), each trained and tested on the same dataset using matched hyperparameters. Boxes show median and interquartile range, whiskers extend to 1.5× the interquartile range, and points represent individual test slides (*n* = 10). A Friedman test detected no significant difference in Dice scores across the five architectures [χ²(4) = 7.76, *p* = 0.101; Kendall’s W = 0.194].

**Figure S2. Pairwise relationships between DUCK-Net coverage and IHC markers across DDC time course.** Scatterplot matrix showing pairwise relationships between DUCK-Net coverage and IHC markers (KRT19, SOX9, EpCAM, α-SMA, PSR). Linear relationships were observed between all variables with no evidence of non-linear patterns.

**Figure S3. Regression model diagnostics for multiple linear regression of DUCK-Net coverage against IHC markers.** Residual diagnostic plots for multiple linear regression model. Orange lines represent LOESS smoothing curves (or the theoretical normal distribution for the Q–Q plot). Residuals versus Fitted (top left): random scatter indicates the linearity assumption is satisfied. Normal Q–Q (top right): points along the diagonal confirm approximately normally distributed residuals. Scale–Location (bottom left): a relatively stable trend line indicates acceptable homogeneity of variance. Residuals versus Leverage (bottom right): no points exceed Cook’s distance thresholds, confirming the absence of highly influential observations.

**Figure S4. Cross-model evaluation of DUCK-Net in CDE and MCD models of liver injury.** Scatter plot of Dice coefficient against DR burden for individual H&E-stained sections from two alternative models of liver injury: CDE diet (purple; *n* = 6) and MCD diet (orange; *n* = 9). The DUCK-Net was trained on the DDC model, applied without re-training, and compared against expert pathologist annotation of DR. DR burden is expressed as annotated DR area as a percentage of total annotated tissue area. Each point represents one animal. Grey line shows linear regression fit with 95% CI.

**Table S1.** Annotated pixel statistics and dataset composition for model development and evaluation.

|  | **Annotated pixels (millions)** | | |  |  |  |
| --- | --- | --- | --- | --- | --- | --- |
|  | Image | Tissue | DR | Image tissue coverage (%) | Tissue DR coverage (%) | Samples used |
| Train | 13,535 | 8,890 | 378 | 65.7% | 4.3% | WSI  50× sample selection |
| Validation | 4,220 | 2,683 | 130 | 63.6% | 4.8% | WSI  50× sample selection |
| Test | 4,420 | 4,072 | 627 | 92.1% | 15.4% | 10× independent sample selection |
| **Total** | **22,175** | **15,645** | **1,135** | **70.5%** | **7.3%** |  |

Pixel counts (millions) are reported for total annotated image area, tissue area, and DR area, calculated from original full-resolution whole-slide images prior to down-sampling. Tissue image coverage (%) indicates proportion of annotated image area identified as tissue, and tissue DR coverage (%) indicates proportion of tissue area annotated as DR. Training data included one WSI split 75:25 into training and validation regions, supplemented with sampled regions from 50 additional WSIs distributed across these sets. The independent test set comprised 10 regions sampled from separate WSIs not used during training or validation.

**Table S2.** DUCK-Net performance metrics for individual test samples.

| **Slide ID** | **Image** | **Dice** | **Precision** | **Recall** | **Specificity** | **Accuracy** |
| --- | --- | --- | --- | --- | --- | --- |
| 131 | 1 | 0.915 | 0.914 | 0.916 | 0.974 | 0.961 |
| 132 | 2 | 0.896 | 0.883 | 0.910 | 0.975 | 0.964 |
| 133 | 3 | 0.893 | 0.875 | 0.912 | 0.985 | 0.978 |
| 134 | 4 | 0.876 | 0.928 | 0.830 | 0.979 | 0.943 |
| 135 | 5 | 0.867 | 0.810 | 0.932 | 0.976 | 0.972 |
| 136 | 6 | 0.780 | 0.673 | 0.927 | 0.952 | 0.949 |
| 137 | 7 | 0.827 | 0.766 | 0.898 | 0.969 | 0.962 |
| 138 | 8 | 0.884 | 0.823 | 0.955 | 0.961 | 0.960 |
| 139 | 9 | 0.768 | 0.901 | 0.670 | 0.991 | 0.958 |
| 150 | 10 | 0.831 | 0.860 | 0.804 | 0.984 | 0.965 |
|  | **Mean** | **0.854** | **0.843** | **0.875** | **0.975** | **0.961** |
|  | **SD** | **± 0.048** | **± 0.074** | **± 0.081** | **± 0.011** | **± 0.009** |

Performance metrics for DUCK-Net segmentation evaluated on 10 independent test samples from mouse liver sections following 21 days DDC dietary injury. Each sample was annotated by a specialist clinical pathologist. Dice coefficient (Dice), precision, recall (sensitivity), specificity, and accuracy are reported for each sample with mean ± SD across the test set.

**Table S3.** Benchmarking of DUCK-Net against established CNN-based segmentation architectures.

| **Test slide** | **U-Net** | **U-Net++** | **DeepLabv3+** | **MA-Net** | **DUCK-Net** |
| --- | --- | --- | --- | --- | --- |
| 131_roi_0 | 0.911 | 0.913 | 0.912 | 0.914 | 0.910 |
| 132_roi_0 | 0.894 | 0.896 | 0.888 | 0.892 | 0.891 |
| 133_roi_0 | 0.900 | 0.904 | 0.898 | 0.899 | 0.892 |
| 134_roi_0 | 0.913 | 0.910 | 0.903 | 0.891 | 0.897 |
| 135_roi_0 | 0.867 | 0.873 | 0.863 | 0.872 | 0.871 |
| 136_roi_0 | 0.739 | 0.739 | 0.755 | 0.747 | 0.757 |
| 137_roi_0 | 0.803 | 0.807 | 0.778 | 0.810 | 0.813 |
| 138_roi_0 | 0.890 | 0.891 | 0.883 | 0.888 | 0.888 |
| 139_roi_0 | 0.789 | 0.790 | 0.790 | 0.817 | 0.782 |
| 150_roi_0 | 0.853 | 0.848 | 0.842 | 0.805 | 0.839 |
| **Test mean ± SD** | **0.856 ± 0.060** | **0.857 ± 0.060** | **0.851 ± 0.057** | **0.854 ± 0.055** | **0.854 ± 0.053** |
| **Best validation Dice** | **0.814** | **0.819** | **0.809** | **0.824** | **0.821** |

Per-slide Dice coefficients on the test set (*n* = 10 slides) for four comparator CNN-based segmentation architectures (U-Net, U-Net++, DeepLabv3+, MA-Net) following re-training using matched hyperparameters. Best Dice scores on the validation set during training are shown in the final row.

**Table S4.** ROC analysis summary for DUCK-Net and IHC markers across clinically relevant comparisons.

| **Comparison** | **Marker** | **AUC** | **95% CI** | **Optimal threshold** | **Sensitivity** | **Specificity** |
| --- | --- | --- | --- | --- | --- | --- |
| **Control versus any injury (Day 0 versus Days 7–35)** | DUCK-Net | 1.00 | 1.00–1.00 | 1.37 | 1.00 | 1.00 |
|  | KRT19 | 1.00 | 1.00–1.00 | 0.94 | 1.00 | 1.00 |
|  | SOX9 | 0.95 | 0.84–1.00 | 1.54 | 1.00 | 0.80 |
|  | α-SMA | 0.82 | 0.65–1.00 | 6.23 | 0.74 | 1.00 |
|  | PSR | 0.97 | 0.90–1.00 | 2.26 | 0.95 | 1.00 |
| **Peak versus early/control (Days 14–21 versus Days 0–7)** | DUCK-Net | 0.98 | 0.94–1.00 | 6.08 | 1.00 | 0.89 |
|  | KRT19 | 1.00 | 1.00–1.00 | 2.47 | 1.00 | 1.00 |
|  | SOX9 | 0.67 | 0.36–0.98 | 3.55 | 1.00 | 0.56 |
|  | α-SMA | 0.90 | 0.75–1.00 | 9.63 | 0.86 | 0.89 |
|  | PSR | 0.98 | 0.94–1.00 | 3.25 | 1.00 | 0.89 |
| **Injury versus Recovery (Days 7–21 versus Days 28–35)** | DUCK-Net | 0.81 | 0.58–1.00 | 5.47 | 0.82 | 0.88 |
|  | KRT19 | 0.66 | 0.38–0.94 | 2.43 | 0.73 | 0.75 |
|  | SOX9 | 0.97 | 0.89–1.00 | 3.72 | 1.00 | 0.88 |
|  | α-SMA | 0.82 | 0.59–1.00 | 4.99 | 1.00 | 0.62 |
|  | PSR | 0.66 | 0.40–0.92 | 3.57 | 0.36 | 1.00 |

ROC analysis was performed to evaluate the discriminatory performance of DUCK-Net and conventional IHC markers (KRT19, SOX9, α-SMA, PSR) for classifying injury states in the DDC model. The AUC was calculated from empirical (non-parametric) ROC curves with 95% CIs estimated using the DeLong method. Optimal classification thresholds were determined by maximising Youden's J statistic (sensitivity + specificity − 1). Sensitivity and specificity are reported at the optimal threshold.

**Table S5.** Time course quantification of DUCK-Net, IHC markers, and interpolated spatial transcriptomic domains.

| **Day** | **Phase** | **DUCK-Net (%)** | **KRT19 (%)** | **SOX9 (%)** | **EpCAM (%)** | **α-SMA (%)** | **PSR (%)** | **LPLC** | **Chol** |
| --- | --- | --- | --- | --- | --- | --- | --- | --- | --- |
| 0 | Baseline | 0.35 ± 0.09 | 0.47 ± 0.12 | 1.24 ± 0.51 | 0.74 ± 0.10 | 3.82 ± 0.90 | 1.44 ± 0.28 | 0.102 | -0.026 |
| 7 | Injury | 4.86 ± 0.82 | 1.88 ± 0.30 | 9.26 ± 2.37 | 2.41 ± 0.54 | 9.28 ± 1.43 | 2.68 ± 0.49 | 0.362 | -0.018 |
| 14 | Injury | 7.53 ± 0.61 | 2.92 ± 0.16 | 6.94 ± 0.79 | 2.72 ± 0.80 | 13.59 ± 2.23 | 4.26 ± 0.34 | 0.514 | -0.007 |
| 21 | Injury | 8.31 ± 0.39 | 3.19 ± 0.53 | 10.90 ± 4.37 | 4.03 ± 0.48 | 13.61 ± 2.56 | 5.45 ± 0.47 | 0.590 | 0.005 |
| 28 | Recovery | 5.51 ± 1.24 | 2.65 ± 0.48 | 3.30 ± 0.74 | 2.97 ± 0.26 | 8.84 ± 2.73 | 5.18 ± 0.67 | 0.626 | 0.000 |
| 35 | Recovery | 3.49 ± 0.38 | 1.80 ± 0.20 | 2.50 ± 0.35 | 2.57 ± 0.15 | 2.79 ± 0.49 | 4.77 ± 0.73 | 0.518 | -0.008 |

Quantification of DUCK-Net coverage and IHC markers across DDC injury and recovery time course. DUCK-Net coverage and IHC markers (KRT19, SOX9, EpCAM, α-SMA) are expressed as percentage positive area or cells; PSR as percentage collagen area. Values shown as mean ± SE (*n* = 4–5 per timepoint). LPLC and Chol domain scores were derived from spatial transcriptomic (Stereo-seq) data from Wu *et al.* [12] and interpolated to match experimental timepoints using linear interpolation.
